# Pregnancy-Related Hysterectomy for Peripartum Hemorrhage: A Literature Narrative Review of the Diagnosis, Management, and Techniques

**DOI:** 10.1155/2021/9958073

**Published:** 2021-07-06

**Authors:** Dimitrios Tsolakidis, Dimitrios Zouzoulas, George Pados

**Affiliations:** 1st Department of Obstetrics & Gynecology, Aristotle University of Thessaloniki, Greece

## Abstract

Postpartum hemorrhage is a life-threatening situation, in which hysterectomy can be performed to prevent maternal death. However, it is associated with high rates of maternal morbidity and mortality and permanent infertility. The incidence of pregnancy-related hysterectomy varies across countries, but its main indications are the following: uterine atony and placenta spectrum (PAS) disorders. PAS disorder prevalence is rising during the last years, mainly due to the increased number of cesarean sections. As a result, obstetricians should be aware of the difficulties of this emergent condition and improve its accurate antenatal diagnosis rates, as well as its modern management strategies. Of course, special skills are required during a pregnancy-related hysterectomy, so these patients should be referred to centers of excellence in antenatal care, where a multidisciplinary team approach is followed. This study is a narrative review of the literature of the last 5 years (PubMed, Cochrane) regarding postpartum hemorrhage to offer obstetricians up-to-date knowledge on this pregnancy-related life-threatening issue. However, there is a lack of available high-quality data, because most published papers are retrospective case series or observational cohorts.

## 1. Introduction

Postpartum hysterectomy is an intervention performed in life-threatening situations to prevent maternal death [1]. Obstetricians face a dilemma: to perform postpartum hysterectomy or to attempt other conservative uterine sparing techniques that may result in severe morbidity or death. Unfortunately, postpartum hysterectomy results in the loss of future fertility and is associated with high prevalence of maternal morbidity and mortality [2]. Historically, it was first performed successfully by Porro in 1871 [3] and become in the 1950s an elective but controversial procedure, due to excessive blood loss and high incidence of urinary tract injuries [4, 5]. Today, it is mainly used as the final step in several postpartum hemorrhage protocols, because blood transfusions and other interventions (e.g., uterotonics) have reduced its need [6].

There is no globally accepted definition that places a limit in the period of time that the hysterectomy is performed, so the definitions vary among published studies. Some authors define peripartum hysterectomy as the hysterectomy performed at the time of delivery, or at any time from the delivery to discharge at the same hospitalization [7], while others as the removal of the uterus during pregnancy or immediate postpartum [8]. When performed at the time of the cesarean delivery, it is defined as cesarean hysterectomy. Another used term is postpartum hysterectomy, when it is performed after the delivery of the fetus within 24 h or 48 h or during the same hospitalization or within 6 weeks [6]. All these different definitions, in combination with the low incidence of hysterectomy associated with pregnancy, make it difficult to compare results between studies [9]. Therefore, the International Network of Obstetric Survey Systems (INOSS) proposed a definition of “pregnancy-related hysterectomy”: surgical removal of the uterus during pregnancy, from 22 weeks of gestation or up to 42 days postpartum [10]. The term includes hysterectomies after cesarean or vaginal delivery. This definition is wide enough to include all possible indications of hysterectomy.

Worldwide, the incidence of pregnancy-related hysterectomies varies widely [7] and is increasing over time (71.6 to 82.6 per 100.000 deliveries) [11]. The main indication for pregnancy-related hysterectomy is severe uterine bleeding that cannot be controlled by conservative measures [12]. The main causes that lead to severe uterine hemorrhage and cesarean hysterectomy are uterine atony, placenta spectrum disorders, uterine rupture, and sepsis [10]. Possible risk factors for pregnancy-related hysterectomy are abnormal placentation, advanced maternal age, high parity, and cesarean delivery in current or previous pregnancies [9, 12]. Many studies have reported a strong association between cesarean sections, placenta pathologies, and pregnancy-related hysterectomy [13–19]. The relative risk for hysterectomy is known to be increased for every additional previous cesarean section (odds ratios: 0.7 to 15.2 from 1^st^ prior to 6^th^ or more cesarean sections) [20].

Furthermore, it is important to state that the adverse events related to pregnancy-related hysterectomy are substantially higher than those from nonobstetric hysterectomy [2, 21]. It is associated with increased peri- and postoperative complications, especially in low-volume centers, where multidisciplinary team management of these cases is not available [21]. The most common complication is bladder injury (9%), followed by ureteral injury, massive hemorrhage, wound dehiscence, and venous thrombosis [2, 22]. Globally, the risk of death in pregnancy-related hysterectomy is 1% compared to 0.04% for nonobstetric hysterectomy [2].

## 2. Postpartum Hemorrhage

Hemorrhage is a significant cause of maternal mortality and is currently responsible for 27% of all maternal deaths worldwide. It is the 4^th^ leading cause of maternal mortality in the United States and the leading cause worldwide. Postpartum hemorrhage (PPH) is defined as a blood loss of >500 ml after vaginal delivery or >1000 ml after cesarean section within 24 h after birth. However, it is well known that estimation of blood loss during delivery is hard and can be inaccurate. So, the American College of Obstetricians and Gynecologists (ACOG) proposed that PPH is defined as a cumulative blood loss greater than 1000 ml or any degree of blood loss that causes signs or symptoms of hypovolemia, such as tachycardia, tachypnea, oliguria, hypotension, dizziness, pallor, or any altered mental status, occurring within 24 h from delivery. Other authors define severe PPH as a blood loss that requires ≥4 blood units.

The timing for the optimal therapeutic dichotomy between conservative management and pregnancy-related hysterectomy has not been yet defined in cases of PPH, and the highly inaccurate visual calculation of blood loss makes it even harder. So, the need to establish some parameters to evaluate or even predict PPH is necessary. The use of shock parameters (blood pressure, heart rate, and urinary output) has been strongly recommended [23].

During the last years, the shock index, grade of shock, and number of packed red blood cells (PRBCs) have been proposed as possible parameters that can predict the volume of blood loss [24–26]. The shock index can be calculated as the ratio of the heart ratio/systolic pressure. The grade of shock is defined by several parameters: systolic and diastolic pressure, heart ratio, urinary output, and respiration. On the other hand, hemoglobin values and coagulation parameters were not reliable to differentiate the severity of blood loss and were also time consuming. Taking the above into consideration, a massive blood transfusion protocol should be adapted and the choice between conservative aggressive (hysterectomy) treatments should be based on hemodynamic parameters and not on laboratory tests [24]. Moreover, a retrospective study from Lee et al. proposed a predictive scoring model for PPH in PAS disorders [27]. The model included maternal old age (<35: 0, ≥35: 1), antepartum bleeding (no: 0, yes: 2), fetal noncephalic presentation (no: 0, yes: 2), placenta previa type (incomplete: 0, complete: 1), placenta location (posterior: 0, anterior: 1), uteroplacental vascularity (no: 0, yes: 2), and multiple lacunae (no: 0, yes: 1). A score of 5/10 has a sensitivity of 81% and a specificity of 77% for prediction of a severe PPH. The negative predictive value was 95.9%, while the positive predictive value was 38.1%.

Currently, the most common cause of PPH and therefore pregnancy-related hysterectomy is uterine atony, but the rate of PAS disorders as a cause for PPH continues to increase [28]. A recent systematic review and meta-analysis [12] which included women worldwide confirmed the data from other studies [29] and showed that PAS disorders could become the leading cause, because of the rising use of uterotonics and the increasing number of cesarean sections. These findings were also presented in two large multinational cohort studies that were published recently: one that included data from the WOMAN trial [7] and the other from INOSS [10]. Both studies included a large number of pregnancy-related hysterectomies (1020 and 1320, respectively) and showed that the main indication of the hysterectomy was uterine atony (35.3%), followed closely by PAS disorders (34.8%) and uterine rupture as the third cause.

Another important issue is the timing of the hysterectomy which differs from the cause of bleeding. When PAS disorders were the cause, the median time was 0.6 h, compared to ≥13 h for uterine atony. Furthermore, PAS disorders were associated with three times higher risk of hysterectomy compared to uterine atony. In addition, cesarean section was found to increase the risk of hysterectomy (fourfold higher odds) as compared to vaginal delivery. These results could be explained by the fact that prenatal diagnosis of PAS disorders lead to a cesarean section and may inevitably require a hysterectomy. Another undeniable fact is that during a cesarean section, the patient is already in the operating room and the uterus is readily accessible, while during vaginal delivery, the obstetricians might try more other conservative methods to control the PPH. Moreover, the number of previous cesarean sections showed an increased risk for pregnancy-related hysterectomy, and every additional operation added a higher chance of maternal morbidity. Older maternal age was also a significant factor for higher risk of hysterectomy, especially in pregnant women over 40 years old, after adjusting for all confounding factors.

## 3. PAS Disorders

### 3.1. Definitions

**Figure 1:**
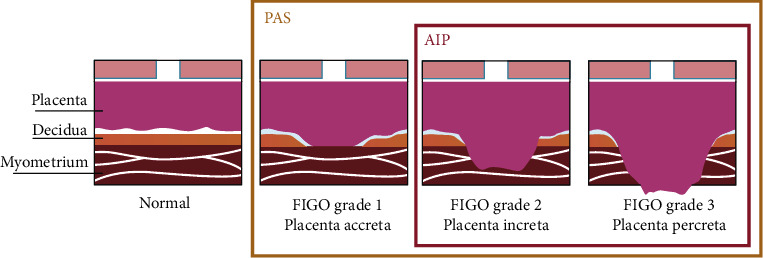
PAS, AIP, and FIGO abnormal placentation definition (from Morlando and Collins [30] with permission).

**Figure 2:**
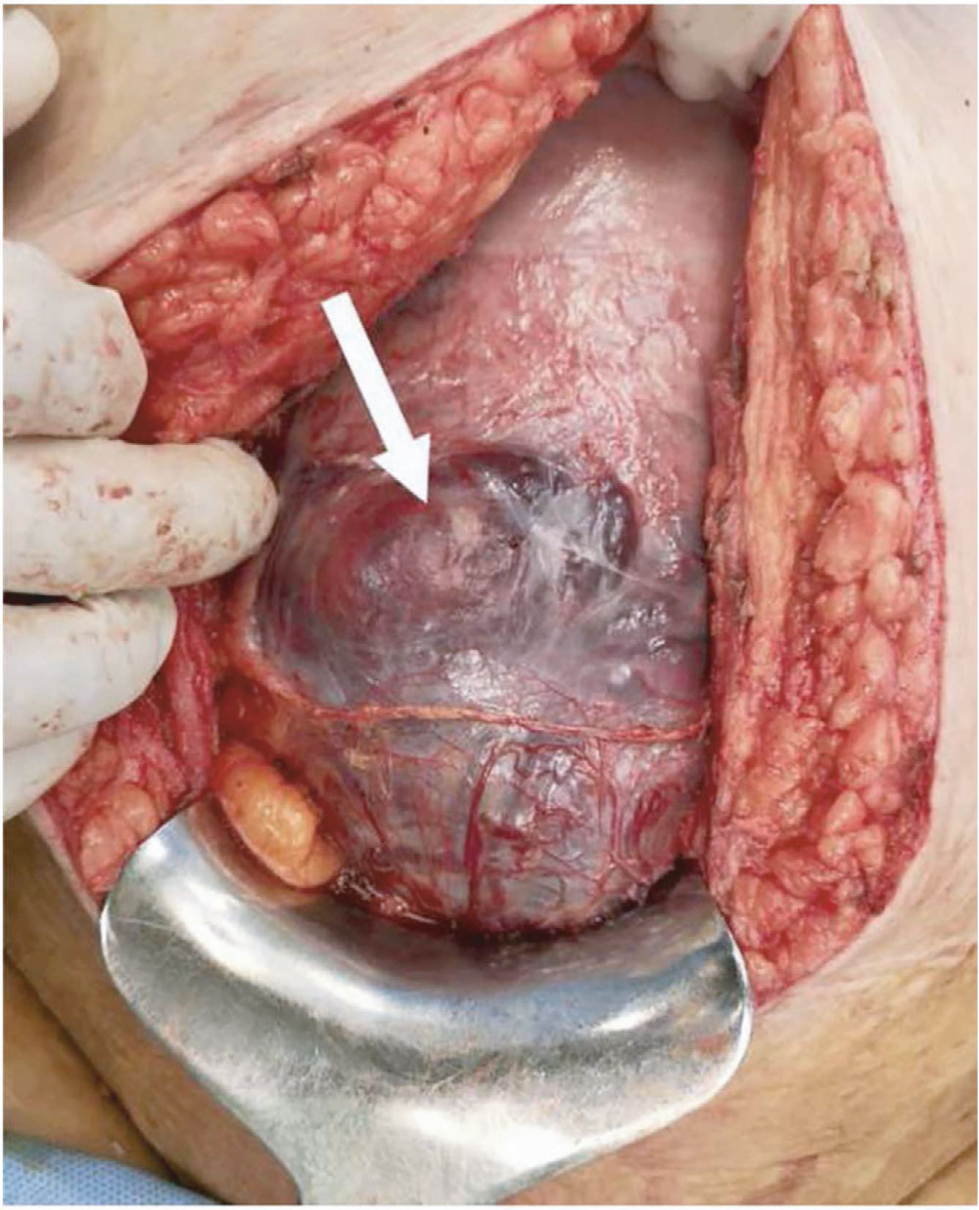
Uterine myometrial dehiscence at 35 weeks, due to prior cesarean sections (from Jauniaux et al. [51] with permission).

PAS is a heterogenous group of disorders, and its definitions vary among literature [30]. The use of the term PAS disorders is a wide term that encompasses the whole spectrum of pathology (Figure 1) and recently has been endorsed by several scientific societies, such as FIGO [31], RCOG [32], ACOG, and SMFM [33]. Based on the depth of the myometrial invasion from the trophoblast, three types of PAS can be categorized [34]: placenta accreta (grade 1) (also referred to as creta, vera, and adherenta), where the chorionic villi attach directly to the surface of the myometrium in the absence of the decidual layer [35]; placenta increta (grade 2), where the chorionic villi penetrate deeply into the myometrium reaching the external layer [36]; and placenta percreta (grade 3), where the chorionic villi invade, reach, and penetrate through the uterine serosa [34, 37]. A clinical-histological grading system has been proposed by FIGO to describe and categorize the different aspects of PAS disorders (Table 1) [38]. The severity of the disorder, hence the possibility of pregnancy-related hysterectomy, increases from placenta accreta to percreta. It is important to be able to recognize other entities-cases that can be easily mistaken as PAS disorders. Firstly, the “uterine window,” which is a dehiscence of the uterine myometrium after a prior cesarean section and the placenta, is visible under the serosa at the time of the operation (Figure 2) [34]. Secondly, there is retention of the placenta during vaginal delivery, when the placenta is separated normally from the uterine wall, in contrast to the PAS disorders, but it is entrapped into the uterus due to the strong or tetanic contraction of the cervix [30].

Another term that is usually used when describing the PAS disorders is placenta previa. This term concerns the positioning of the placenta [39]. Placenta previa develops in the lower segment of the uterus, instead of the upper one, and it is classified according to the relationship/distance of the lower placenta edge from the internal os of the uterine cervix. Definition of minor placenta previa is when the lower edge lies inside the lower uterine segment down to the internal os, and that of major placenta previa is when the placenta covers the uterine cervix. Both are further subdivided into two categories: minor placenta previa to low-lying placenta when the lower placenta edge does not reach the internal os and marginal placenta previa when it does. Major placenta previa is described as partial or complete depending on the amount of the placenta covering the cervix.

### 3.2. Pathophysiology

Many theories have been proposed about how PAS disorders can occur. The one that prevails is that iatrogenic defect of the endometrium-myometrium interface leads to a failure of normal decidualization at the site of the uterine scar and therefore enables abnormally deep trophoblastic infiltration [37]. The extent of the infiltration of the villous tissue inside the myometrium is likely to be connected with the extent of the deciduo-myometrial damage. Another mechanism that has been suggested is that during IVF cycles, a characteristic hormonal milieu at the time of the implantation and placentation may promote deep trophoblast invasion that resulted in PAS [40]. This could be explained either from elevated serum estrogen levels at the time of the implantation, enabling excessive trophoblastic invasion deep inside the myometrium, or from lower serum estradiol levels together with the presence of the thinner decidualized endometrium [41]. Last but not least, one additional theory is based on the finding of unusual uteroplacental vasculature, meaning physiological changes in larger arteries deep in the myometrium, when abnormal invasive placentation is present, compared to normal pregnancies [42].

### 3.3. Risk Factors

Epidemiological studies have shown a strong association between the incidence of PAS disorders, cesarean section rates, and prior cesarean section numbers [36]. Globally, over the last 40 years, the rate of cesarean deliveries has risen from 10% to 40%, and at the same time, there has been a 10-fold increase in the incidence of PAS disorders [31]. Moreover, increased cesarean sections have increased the incidence of placenta previa [43]. The incidence of PAS disorders also increases with every prior cesarean delivery: from 4.5% for one up to 44.9% for four or more cesarean sections, compared to vaginal deliveries [44]. Similarly, the risk of PAS disorders in women with a placenta previa and prior cesarean section was 3%, 11%, 40%, 61%, and 67% for the first, second, third, fourth, and fifth or more cesarean sections, respectively [20]. Current data suggest that over 90% of women diagnosed with any PAS disorder also have a placenta previa [45]. The combination of these two pathologies leads to high maternal morbidity and mortality due the severe postpartum hemorrhage [46, 47]. The maternal mortality has been reported in some studies as high as 7%, when placenta previa with percreta is present [48].

Other risk factors except placenta previa, cesarean section, and prior cesarean section numbers are any procedure that causes surgical damage to the uterine wall integrity [36, 37]. Specifically, operative hysteroscopy, suction curettage, surgical termination, and endometrial ablation have been reported to cause later PAS disorders to nulliparous women (no other risk factor) [37, 49]. Cases of PAS can occur even after myomectomy, but with a relative lower risk [50]. Finally, studies from the later years have shown that IVF, especially with cryopreserved embryos, increases 4- to 13-fold the risk of PAS disorders [51].

### 3.4. Diagnosis

Accurate antenatal diagnosis of PAS disorders is essential for the maternal morbidity and mortality. A false-negative antenatal diagnosis may lead to a routine low transverse uterine incision and a massive placental blood loss, even before the fetus is delivered. On the other hand, a false-positive diagnosis will result in an unnecessary midline skin incision and a fundus uterine incision, which increases the risk of intra- and postoperative complications [45]. It is of high importance that these cases should be diagnosed on time and referred to a center of excellence, where a multidisciplinary team (MDT) approach is available. These centers of excellence are less likely to require an emergency surgery, large-volume transfusion protocols, and reoperation within 7 days from delivery for any complication, compared to centers with no standardized management protocol [52–54]. A recent systematic review and meta-analysis confirmed that maternal outcomes have improved over time with increasing experience within a center of excellence with the MDT approach performing 2-3 cases per month [55]. Some criteria have been proposed for these centers of excellence (Table 2) [53]. Prenatally unsuspected PAS disorders are usually associated with higher risk of severe PPH, due to the repeatedly attempts of the surgeons to remove the placenta from the uterine wall [56]. When the placenta is left in situ, because it was antenatally diagnosed, there is less blood loss and less need for transfusion [57]. These findings were confirmed from a recent systematic review and meta-analysis, which showed that antenatal diagnosis of PAS reduces perioperative complications and especially surgical bleeding [58]. The antenatal diagnostic accuracy of PAS is between 90 and 95% in several studies [45, 59, 60], especially in experienced centers. However, recent population studies show that PAS disorders remain undiagnosed until delivery in half [57, 59] to two-thirds [61] of the cases in the overall population.

### 3.5. Ultrasound

Ultrasound imaging during pregnancy is considered highly accurate in the detection of PAS disorders, when it is performed by a skilled operator [32]. A recent systemic review and meta-analysis found a pooled sensitivity of 88% and 97% in retrospective and prospective studies, respectively [45]. Numerous techniques have been added to the grayscale ultrasound, such as color Doppler and 3D power Doppler, to improve the sensitivity of the exam [45, 62]. Over the years, many studies investigated the predictive value of several signs for PAS, and their performance has shown considerable variability [63]. This could be attributed to the limitations of these studies and the different terminology reported: the same sign described using different names or the same term for different findings [30]. Another additional problem is the fact that the diagnostic technique relies strongly on the opinion of the operator, according to his experience and training [64]. Other factors that can alter the ultrasound signs are the scanning conditions (e.g., too full or too empty bladder), ultrasound equipment, and gestational age [30]. One important problem, which still remains, is the lack of sign or combination of signs that can effectively predict the depth of myometrial invasion from the trophoblast [34, 45, 65]. Recently, the European Working Group on Abnormal Invasive Placenta (EW-AIP) has proposed some standardized descriptions of ultrasound signs associated with PAS disorders (Table 3) [64].

### 3.6. Magnetic Resonance Imaging (MRI)

Although ultrasound is the first-line imaging tool for the screening and diagnosis of PAS disorders, the role of MRI has been well established for the diagnosis of PAS, with high sensitivity and specificity [66]. Both MRI and ultrasound have comparable predictive parameters, and no superiority has been demonstrated [66, 67]. It is unclear if MRI can improve the diagnosis for PAS compared to what can be achieved from an experienced ultrasound operator [32, 33]. MRI may be less operator-dependent, but the high cost and the limited access to equipment and an expert radiologist make it impractical as a screening tool [68]. Moreover, a recent study found that MRI resulted in a change in diagnosis that could alter clinical management of PAS in more than one-third of cases, but, when changed, the diagnosis was often incorrect [69]. So, it is recommended that the first screening is performed by ultrasound, and if a suspicion of PAS is raised, then an MRI should be proposed as a second-line imaging tool [30]. It helps to assess the depth of myometrial invasion and parametrial involvement [32]. Another advantage is that MRI can overcome certain technical limitations of the ultrasound in the diagnosis of PAS: unfavorable placenta location (posterior) or high maternal BMI, and the entire pelvis can be easily studied and reevaluated by different physicians [30]. The International Society for Abnormally Invasive Placenta (IS-AIP) has recently proposed standardized definitions of the MRI descriptors (Table 4) [70].

### 3.7. Clinical Criteria

The ultimate confirmation of PAS should be performed peripartum, before any surgical treatment. There is no established clinical diagnostic method; therefore, surgeons should be aware of all possible predictive clinical signs [53, 71–75]: difficult manual or piecemeal removal of the placenta, absence of placenta separation 20-30 min after delivery (despite active management with bimanual uterine massage, umbilical cord traction, and use of oxytocin), retained placenta fragments requiring curettage (vaginal delivery), and severe bleeding from the placenta bed after its removal (cesarean section). However, some basic steps have been proposed during laparotomy for the diagnosis of PAS [76]. (a) The external surface of the uterus and the pelvis was inspected for abnormal appearance of the serosa over the placental bed (bluish/purple appearance) with evident distension (placental bulge) or obvious invasion through the uterine surface. (b) If there is no evidence of the most severe PAS disorders, a uterine incision, leaving the placenta intact, should be performed and gentle cord traction should be attempted. If the uterine wall is pulled towards the direction of the traction with no placenta separation (“dimple” sign) and there is apparent contraction of the uterus separating from the placenta bed, then PAS can be diagnosed. (c) If the previous two steps do not reveal PAS, then a gentle digital exploration can be attempted to assess the presence of a cleavage plane between the uterus and the placenta.

### 3.8. Histopathological Criteria

The histopathological diagnosis of PAS can be very difficult, because of the surgeons' attempts to remove the placenta from the uterus and the fact that when conservative management is attempted, the whole placenta is left in situ [51]. The main histopathological criterion used for the diagnosis is the absence of decidual/Nitabuch layer between the tip of anchoring villi and superficial myometrium [77]. Nevertheless, this criterion can be elusive and simplistic, because these areas can be found at placentas in advanced gestational age pregnancies, without the presence of PAS disorders [34]. When PAS disorders are found during the histopathological examination, it is of high importance to confirm and report the depth of villous invasion of the uterine myometrium, in order to differentiate the types of PAS [51]. But often enough, there is a lack of clear description of the histological criteria used to define the different types/grades of PAS [65, 78].

### 3.9. Biomarkers

Several possible biomarkers have been proposed and tested for years in different studies for the diagnosis of PAS, depending on the gestational age [79]. When PAS disorders are present, *β*-hCG was lower and PAPP-A higher, compared to normal pregnancies, at 11-12 weeks of gestation, while at 14-22 weeks of gestation, serum levels of *β*-hCG and AFP were higher [30]. Currently, there is no effective established biomarker for a serological screening of PAS [62].

## 4. Management Strategies: Hysterectomy

The majority of the surgeons that are experts on pregnancy-related hysterectomy prefer to leave the placenta in situ and perform a primary cesarean hysterectomy at delivery [80–83]. However, some authors in the literature opt for conserving treatment for PAS disorders and leave the placenta in the uterus with the expectation of spontaneous placenta absorption or delivery [84]. This conservative management may decrease the maternal morbidity of the pregnancy-related hysterectomy, but there are several complications that accompany this treatment plan: massive hemorrhage, infection, sepsis, disseminated intravascular coagulation, and ultimately hysterectomy [85, 86]. Questions have been raised about the high prevalence of PAS in recent population studies and the even higher successful rates of conservative treatment, which could be a misleading conclusion, due to the wrongful inclusion of no PAS placenta retention or/and uterine dehiscence in their data [51]. A recent systematic review [87] on conservative treatment for PAS disorders revealed high maternal morbidity during conservative treatment for placenta percreta, which is one of the main reasons that pregnancy-related hysterectomy is not preferred as a first-choice treatment. Another systemic review and meta-analysis stated that conservative management failed to prevent a secondary hysterectomy in the majority of the cases of previa PAS [45]. Overall, there are no RCTs or well-designed prospective observational studies comparing hysterectomy and conservative treatment for the same type of PAS disorder. Any attempt for conservative treatment should be made in large and experienced centers, where surgery could be performed in a 24 h manner from experienced surgeons [88].

The steps for pregnancy-related hysterectomy are the same as those for nonobstetric hysterectomy [6]:

1. Separation of the round ligament
2. Separation of the broad ligament
3. Dissection of the bladder and perivesicular space
4. Palpation, clamping, and separation of the cardinal ligament and uterine arteries
5. Separation of the uterosacral ligament
6. Closure of the vaginal cuff

However, the changes that occur to the female reproductive system during pregnancy may further complicate the procedure [6]. The main difficulty is the increased uterine blood flow, especially during late gestation. There is a 10- to 30-fold increase in the uterine blood flow from the beginning to the end of pregnancy [89, 90]. Tissue fragility and edema are also increased, which makes handling tissue more difficult. The enlarged uterus makes its manipulation and overall visualization of the pelvis much harder, and the normal anatomical relationships and structures are often displaced. The closest important structures that should be recognized and preserved are the ureters which are tortuous and distended and with significant hydroureter. Completion of pregnancy-related hysterectomy can be either total or subtotal, without the removal of the cervix. The goal should be total hysterectomy, because of the potential risk of malignancy developing in the cervical stump and the need for regular cervical cytology, and most of the times, the cervix is the cause of postoperative bleeding (placenta previa PAS) [91]. But when its removal may compromise the hemostasis, it should be left in place. The ovaries should be reserved, but the fallopian tubes should be removed to reduce ovarian cancer risk.

The position of the patient is either supine or dorsal lithotomy, and the incision is based on the expected difficulty and complications, either vertical or extended transverse. Preoperative prophylactic antibiotics should be administrated, to reduce surgical site infection, which was confirmed for a Cochrane review where the use of antibiotics reduced the risk of wound infection, endometritis, and serious maternal infection [92]. Large-bore venous access and pneumatic compression devices are recommended, and central venous access should be considered [56]. In a randomized trial involving women with severe PPH, it showed that the use of tranexamic acid reduced the risk of death due to blood loss [93]. One study compared the surgical outcome with or without LigaSure during pregnancy-related hysterectomy and found that its use resulted in less operative time, less blood loss, and reduced incidence of severe PPH [94]. Moreover, these women are at increased risk for postpartum venous thromboembolism, due to their long and complex surgery and their immobilization [56].

### 4.1. Hysterectomy for PAS Disorders

The increasing incidence of PAS disorders and the high maternal morbidity during hysterectomy for abnormal placentation make the management strategy of this pathology very difficult. There are no RCTs or high-quality studies for the management of PAS disorders, and the only available data are from retrospective cohort studies and case series. As a result, different strategies have been proposed from several authors.

The cornerstone for the management of PAS disorders is to avoid any attempt to remove the placenta from the uterine wall [30]. Making no attempt decreases hemorrhage and blood transfusion [57]. Recommendations [30] to avoid intercourse and cervical examination are of unproven efficacy, but these measures might have some meaning in cases of placenta previa. Bed rest is recommended in women with bleeding, but it is also unknown if it affects the outcome of the pregnancy. Early elective cesarean section may reduce the risk of bleeding [95], but it increases the risk of neonatal prematurity. So, it is of high importance to define the best time for delivery in women with PAS disorders. Planned delivery ranges from 34 to 38 weeks [30]. Delivery until after 36 w+0 d could be offered to women with no history of prior preterm birth, no vaginal bleeding, no preterm premature rapture of membranes (PPROM), and no uterine contractions. On the other hand, delivery around 34 w+0 d could be offered to women with any of the above-mentioned problems. 34-35-week pregnancy-related hysterectomy with the placenta left in situ is recommended by ACOG [33]. Antenatal steroid prophylaxis should be administrated to the mother (12 mg of betamethasone intramuscularly from 2 to 7 days before delivery and repeated 24 h later) in order to enhance fetal pulmonary lung maturity [96].

Pregnancy-related hysterectomy is the gold standard for the treatment of PAS disorders. However, this radical approach is associated with high rates (40-50%) of severe maternal morbidity, especially hemorrhage and trauma to the surrounding organs, and a 7% rate of maternal mortality [97, 98]. These figures are improved significantly when women with PAS are referred to centers of excellence, where the MDT approach is available and rates of 0.05% of mortality are achievable [78]. General and regional anesthetic techniques can be safely offered to these women [32], but always there might be a need to convert from regional to general during the procedure [91]. Most surgeons prefer a vertical skin incision to allow adequate access to the uterus (especially when the placenta is anterior and towards the level of the umbilicus) and the pelvic wall, while some opt for a large transverse incision, like a modified Maylard incision, for faster healing and better cosmetic result [30, 51], but there is insufficient data of its use in the management of PAS disorders [91]. The uterine incision should always avoid the placenta, so in many cases, a fundal incision is chosen. Intraoperative ultrasound could help to identify the upper edge of the placenta and safely guide the hysterotomy for the delivery of the fetus [99]. Pregnancy-related hysterectomy for PAS disorders is rather technically challenging, compared to hysterectomy for uterine atony, due to high risk of adjacent organ damage [100]. Urinary tract injuries are described in 29% of the procedures, with a reported rate of 76% for bladder lacerations, 17% for ureteral injuries, and 5% for genitourinary fistulas [101]. Injuries to other abdominal organs are less common [102]. The main risk factors for these injuries are depth and extension of placenta invasion, intraoperative blood loss, and the number of prior cesarean sections [103].

Another proposed scenario is the delayed hysterectomy, instead of primary radical surgery [30]. After the delivery of the fetus, the uterus is closed with the placenta left in situ, and the maternal abdomen is also closed. Then, a planned hysterectomy is performed 3-12 weeks postpartum [32]. This approach has the rationale that the uterine perfusion and vascularity are reduced, even with the placenta in situ, so the delayed hysterectomy is less risky. This scenario is an option during an emergency pregnancy-related hysterectomy, when the surgeon has limited experience at this complex surgical procedure [30].

Although uterine stent placement can be beneficial in preventing ureteral injury and intraoperative complications, there is no strong evidence in order to routinely recommend them to all PAS cases [76, 101]. So, their placement should be individualized based on the depth and lateral extent of the invasive placentation. Prophylactic endovascular balloon catheters have been proposed as a method to reduce intraoperative blood loss during pregnancy-related hysterectomy, in order to improve maternal morbidity and also allow the surgeon to operate in a “cleaner”—improved visibly—field. However, their use is controversial, mainly because of their high possible complication rates, such as vessel rapture, thromboembolism, risks for damage of pelvic structures, and disturbance of blood supply to the lower limbs [30, 104, 105]. Furthermore, PAS is associated with extensive aberrant neovascularization, and the occlusion of some pelvic vessels may lead to increased blood loss from the collateral vessels [30]. In addition, two RCTs comparing the placement of balloon catheters in the iliac arteries with no intervention at all found no difference in the number of PRBCs transfused to the patients [106, 107], and a recent RTC comparing bilateral internal iliac artery ligation versus controls found no difference regarding intraoperative blood loss [108].

The main goal during pregnancy-related hysterectomy for PAS is to minimize surgical blood loss. During the procedure, especially for previa percreta, some key steps can be recognized [109, 110]. Each of these steps takes time, and the total skin-to-skin duration can take 2-3 hours for experienced surgical teams:

1. Intra-arterial occlusion balloon catheter placement

This is not always necessary. A balloon is placed in both common iliac arteries [111]. The balloon is inflated at the ligation of the upper uterine artery branch or when the bladder separation is started. However, the balloon occlusion should be within 40 min.

(2) Ureter stent placement

This is not always necessary. When needed, they should be placed in the operating theater, just before surgery. There is a risk of emergency surgery, due to uterine contractions, when the stents are placed the day before the surgery.

(3) “Holding the cervix” technique

**Figure 3:**
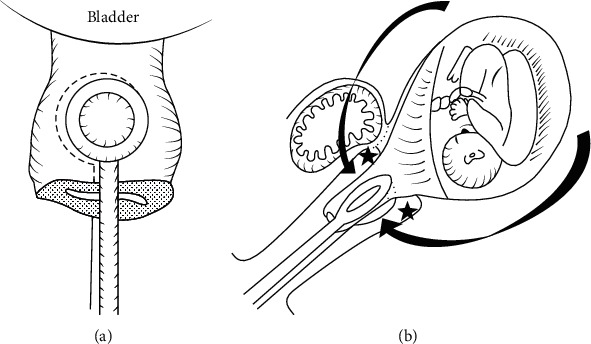
Holding the cervix: (a) anterior-posterior view; (b) lateral view (from Matsubara et al. [109] with permission).

The uterine cervix is closed with round forceps, simultaneously over the anterior and posterior cervical lip (Figure 3) [112, 113]. This has two advantages: firstly, there should be better evaluation of bleeding over time, because without the occlusion of the cervix, the blood flowed to the vagina and the operating field looked falsely dry. Secondly, the metal consistency of the forceps clearly indicates the site to be transected, because pregnancy makes the tissue of the uterus soft and hard to identify the vaginal transection site.

(4) Midline access and hysterotomy

Midline skin incision extended from 2 cm above the pubic bone to 3-4 cm below the umbilicus. Hysterotomy usually towards the fundus vertically, avoiding the placenta, for the delivery of the fetus minimizes blood loss before the delivery.

(5) Avoiding uterotonic agents

Uterotonic agents should not be used, because they might cause partial placenta separation, leading to severe PPH at the beginning of the hysterectomy.

(6) Superior devascularization (M cross double ligation)

**Figure 4:**
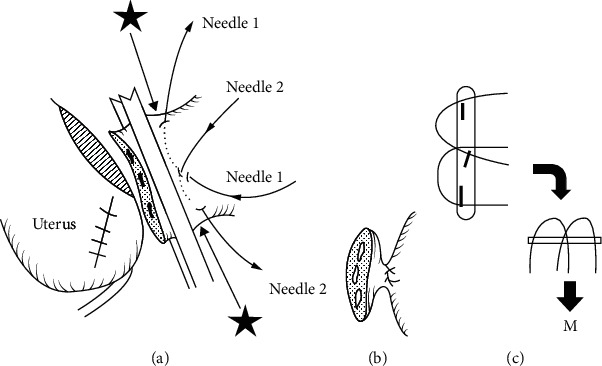
M cross double ligation for the ovarian ligament (from Matsubara et al. [109] with permission).

Release and ligation of the round ligaments and utero-ovarian bilateral pedicle (Figure 4) are performed. At this stage, the risk of bleeding is from excessive upward traction of the uterus from the lateral straight clamps.

(7) Retroperitoneal Dissection

**Figure 5:**
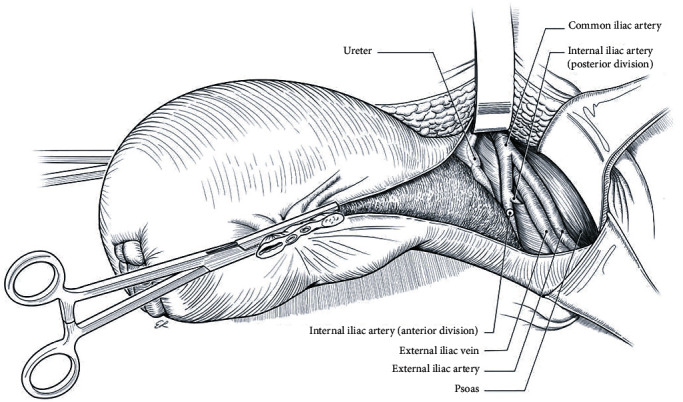
Retroperitoneal devascularization (from Kingdom et al. [110] with permission).

Skeletonization of the uterus down to the cardinal ligaments and opening of the paravesical spaces occur. It also includes a cephalad pelvic sidewall dissection, medially of the psoas muscle to locate the common iliac artery and the external iliac vein and artery. Then, there is exposure of the internal iliac artery and medially the ureters, where ureterolysis is performed (establishing a safe distance between the ureter and the cardinal ligament) (Figure 5). An alternation of this step is the ligation of the internal iliac arteries, 3-5 cm distal from their separation. The exposed arteries could be either sutured or left with a suture loop, for later rapid ligation if severe hemorrhage occurs. Aortograms showed that the low immediate effectiveness of this ligation was substantially diminished by the presence of other pelvic anastomoses [114]. As a result, any clinical benefit in blood loss from this intervention is only short-term and less than 20 minutes in duration.

(8) Bladder Dissection

**Figure 6:**
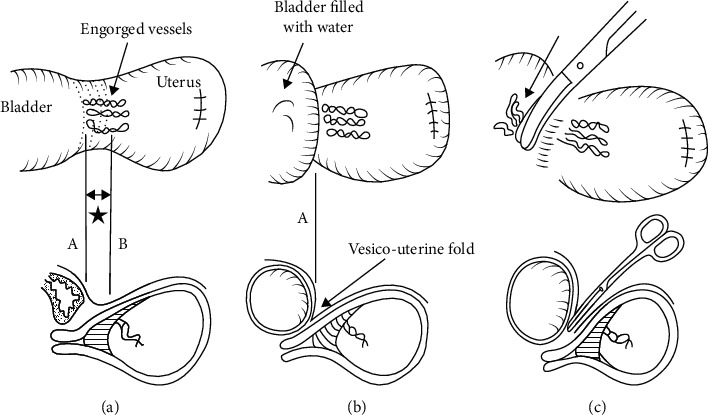
Bladder wall dissection, with filling the bladder (from Matsubara et al. [109] with permission).

**Figure 7:**
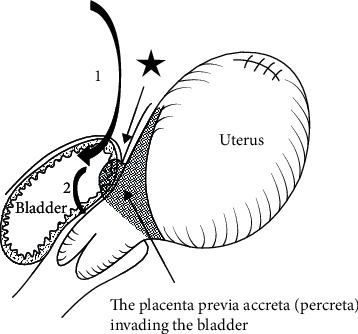
Intentional bladder opening (from Matsubara et al. [109] with permission).

This step is prolonged compared to the nonobstetric hysterectomy, up to 30-40 minutes. At this step, severe hemorrhage may occur. In most women, due to prior cesarean sections, the bladder top is located more cephalad than normal, therefore tempting the surgeon to start the dissection higher (line B at Figure 6) to avoid bladder injury, traumatizing the engorged vessels, causing severe hemorrhage. Cautious lateral to medial dissection is performed, including dividing the engorged blood vessels and adipose layer down with the bladder. Filling the bladder with 100-300 ml of methylene blue could help identify the superior bladder wall margin (line A at Figure 6). This helps to identify the engorged vessels and carefully avoid them (Figure 6). In case of bladder invasion from the placenta, this step is modified, and intentionally cystotomy is performed with resection of the affected portion of the posterior wall of the bladder en bloc with the uterus, followed by bladder repair (Figure 7) [115]. In case of parametrial placenta invasion, extensive retroperitoneal dissection might be required, in order to achieve hemostasis, or a subtotal hysterectomy could be performed [116].

(9) Colpotomy

**Figure 8:**
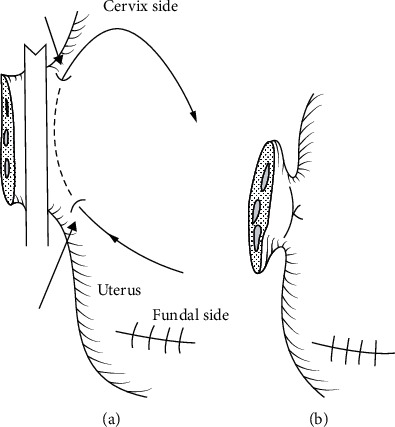
Double distal edge pickup (from Matsubara et al. [109] with permission).

This step also has a high risk of severe hemorrhage. Adequate exposure for the vault entry is created, the main uterine artery pedicles are ligated, and the vaginal angles are secured. The uterine side should remain clamped or ligated using “double distal edge pickup”, to avoid blocking the view from the forceps (Figure 8). Then, colpotomy is performed, and the uterus is removed. The incised edges are clamped incrementally as the vault is opened, in order to minimize blood loss from the margins, followed by suturing of the vault.

**Figure 9:**
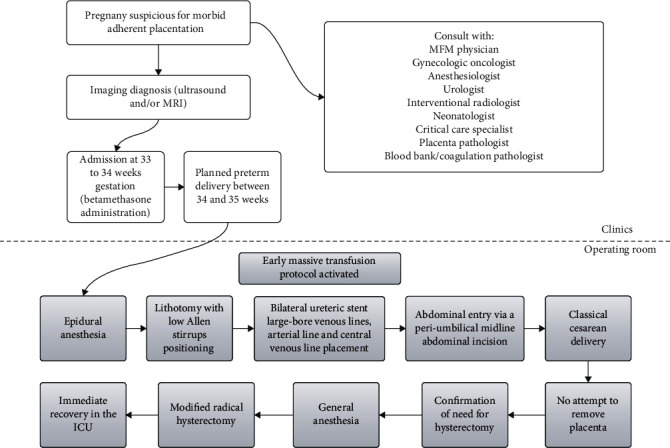
Multidisciplinary protocol for PAS disorders (from Shamshirsaz et al. [52] with permission).

Last but not least, it is of high importance that centers which treat patients with PAS disorders follow standardized protocols with a multidisciplinary strategy (Figure 9). This protocol should include pre-, intra-, and postoperative information about the treatment plan of patients with PAS disorders [52]. Briefly, patients should be admitted at 33-34 weeks of gestation, and a planned hysterectomy should be performed at 34-35 weeks of gestation. All referred patients should undergo an ultrasound examination to confirm the diagnosis, and in cases of lateral or posterior placentation, MRI might be considered. Combined spinal-epidural anesthesia should be offered, and if needed, ureteral stents can be placed. Before induction of general anesthesia, large-bore venous lines, an arterial line, and a central venous line should be placed. All patients receive underbody and overbody forced air warming plus warmed intravenous infusions. Patients are in lithotomy position with low Allen stirrups to allow visualization of the vaginal bleeding. An abdominal entry is created through a periumbilical midline incision and exteriorization of the pregnant uterus, to allow a fundal or posterior hysterotomy, avoiding the placenta. The placenta is left in situ, without any attempt of removal. 500 ml albumin 5% was administered, before the start of the hysterectomy, because acute volume expansion with colloid reduces intraoperative crystalloid requirement and facilitates hemodilution before hemorrhage [117]. A modified radical hysterectomy technique, which includes ureterolysis, should be performed, with extensive use of a bipolar cautery device (LigaSure). This technique ensures wide enough margins from the friable uterine wall and its fragile vessels. The retroperitoneum should be accessed lateral to the round ligament, exposing the iliac vessels and the ureters. The ovaries can be preserved, but the fallopian tubes should be removed. Ureterolysis should be performed to protect the ureters and allow step-by-step devascularization of the lower segment of the uterus. The engorged vessels between the bladder and the uterine lower segment can be identified and cauterized much easier after the exposure of the lateral anatomy. In cases of deep placenta invasion of the bladder, cystotomy and bladder repair should be preferred instead of persistent attempts of bladder dissection, minimizing blood loss. During hemorrhage, early blood product replacement, using a massive transfusion protocol (PRBCs and frozen plasma in a 1 : 1 ratio), should be encouraged, and electrolyte, ionized calcium, and potassium levels should be measured. In cases of acute-severe hemorrhage, complete laboratory tests can be drawn every 20 minutes. Postoperatively, all patients should receive immediate recovery in the ICU after the operation.

## 5. Conclusions

Hysterectomy is an uncommon procedure for obstetric patients; however, it is the final step of every PPH management protocol. Pregnancy-related hysterectomy might have the same surgical steps as a nonobstetric hysterectomy, but special knowledge is needed in order to prevent severe hemorrhage. It is a life-saving procedure, but with substantial maternal morbidity and mortality. It is of high importance for healthcare professionals to understand that a multidisciplinary management strategy is needed in order to successfully perform this type of hysterectomy, but all obstetricians should know the basic steps and possible complications during a pregnancy-related hysterectomy, in order to successfully perform one in an emergency case. The commonest indications of pregnancy-related hysterectomy are uterine atony, followed closely by PAS disorders. PAS is showing a rapid increase in the last decades, and given the increased rates of cesarean sections, its incidence is likely to increase even more over time. Therefore, physicians should be aware of this pathology and its difficulties in diagnosis and management.

Furthermore, it should be mentioned that the surgical steps—tips and generally the management algorithms—proposed in this narrative review of the literature are based on low-quality studies (mainly retrospective case series), but from high-quality centers with a multidisciplinary approach. The aim of this study was to offer up-to-date knowledge about the latest data on the management of pregnancy-related hysterectomy, but obstetricians should remember that surgical steps might differ from center to center and a risk of bias is possible. Future studies should focus on the collection of high-quality data from well-designed prospective studies on diagnosis (antenatal imaging) and a multidisciplinary team approach for the management strategy.

## Figures and Tables

**Table 1:** Clinical and histological grading system for PAS disorders (FIGO guidelines).

Grade	Definition	
Clinical criteria		Histologic criteria
1. Abnormally adherent placenta (accreta)	At vaginal delivery: no separation with synthetic oxytocin and gentle controlled cord traction; attempts at manual removal of the placenta result in heavy bleeding from the placenta implantation site requiring mechanical or surgical procedures	Microscopic examination of the placental bed samples from the hysterectomy specimen shows extended areas of absent decidua between villous tissue and myometrium with placental villi attached directly to the superficial myometrium. The diagnosis cannot be made on just delivered placental tissue or on random biopsies of the placental bed
	If laparotomy is required (including for cesarean delivery): the same as above; macroscopically, the uterus shows no obvious distension over the placental bed (placental “bulge”), no placental tissue is seen invading through the surface of the uterus, and there is no or minimal neovascularity	

2. Abnormally invasive placenta (increta)	At laparotomy: abnormal macroscopic findings over the placental bed: bluish/purple coloring and distension (placental “bulge”); significant amounts of hypervascularity (dense tangled bed of vessels or multiple vessels running parallel craniocaudally in the uterine serosa); no placental tissue seen to be invading through the uterine serosa; gentle cord traction results in the uterus being pulled inwards without separation of the placenta (so-called the dimple sign)	Hysterectomy specimen or partial myometrial resection of the increta area shows placental villi within the muscular fibers and sometimes in the lumen of the deep uterine vasculature (radial or arcuate arteries)

3. Abnormally invasive placenta (percreta)		
3a. Limited to the uterine serosa	At laparotomy: abnormal macroscopic findings on the uterine serosal surface (as above) and placental tissue seen to be invading through the surface of the uterus; no invasion into any other organ, including the posterior wall of the bladder (a clear surgical plane can be identified between the bladder and uterus)	Hysterectomy specimen showing villous tissue within or breaching the uterine serosa
3b. With urinary bladder invasion	At laparotomy: placental villi are seen to be invading the bladder but no other organs: clear surgical plane cannot be identified between the bladder and uterus	Hysterectomy specimen showing villous tissue breaching the uterine serosa and invading the bladder wall tissue or urothelium
3c. With invasion of other pelvic tissue or organs	At laparotomy: placental villi are seen to be invading the broad ligament, vaginal wall, pelvic sidewall, or any other pelvic organ (with or without invasion of the bladder)	Hysterectomy specimen showing villous tissue breaching the uterine serosa and invading pelvic tissues/organs (with or without invasion of the bladder)

From Jauniaux et al. [38] with permission.

**Table 2:** Criteria for centers of excellence for PAS disorders.

1. Multidisciplinary team
a. Experienced maternal-fetal medicine physician or obstetrician
b. Imaging experts (ultrasound and MRI)
c. Pelvic surgeon (i.e., gynecologic oncology or urogynecology)
d. Anesthesiologist (i.e., obstetric or cardiac anesthesia)
e. Urologist
f. Trauma or general surgeon
g. Interventional radiologist
h. Neonatologist
2. ICU and facilities
a. Interventional radiology
b. Surgical or medical ICU (24-hour availability of intensive care specialists)
c. Neonatal ICU (gestational age appropriate for neonate)
3. Blood services
a. Massive transfusion capabilities
b. Cell saver and perfusionists
c. Experience and access to alternative blood products
d. Guidance of transfusion medicine specialists or blood bank pathologists

From Silver et al. [53] with permission.

**Table 3:** Ultrasound sign definitions for PAS disorders.

US finding	EW-AIP definition
2D grayscale	
Loss of “clear zone”	Loss, or irregularity, of the hypoechoic plane in the myometrium underneath the placental bed (“clear zone”)
Abnormal placental lacunae	Presence of numerous lacunae including some that are large and irregular (Finberg grade 3), often containing turbulent flow visible on grayscale imaging
Bladder wall interruption	Loss or interruption of the bright bladder wall (hyperechoic band or “line” between the uterine serosa and bladder lumen)
Myometrial thinning	Thinning of the myometrium overlying the placenta to <1 mm or undetectable
Placental bulge	Deviation of the uterine serosa away from the expected plane, caused by abnormal placental tissue into neighboring organ, typically bladder; uterine serosa appears intact, but outline shape is distorted
Focal exophytic mass	Placental tissue seen breaking through the uterine serosa and extending beyond it; most often seen inside the filled urinary bladder

2D color Doppler	
Uterovesical hypervascularity	Striking amount of color Doppler signal seen between the myometrium and posterior wall of the bladder; this sign probably indicates numerous closely packed tortuous vessels in that region (demonstrating multidirectional flow and aliasing artifact)
Subplacental hypervascularity	Striking amount of color Doppler signal seen in the placental bed; this sign probably indicates numerous closely packed tortuous vessels in that region (demonstrating multidirectional flow and aliasing artifact)
Bridging vessels	Vessels appearing to extend from the placenta, across the myometrium and beyond the serosa into the bladder or other organs; often running perpendicular to the myometrium
Placental lacuna feeder vessels	Vessels with high-velocity blood flow leading from the myometrium into placental lacunae, causing turbulence upon entry

3D ultrasound+power Doppler	
Intraplacental hypervascularity	Complex, irregular arrangement of numerous placental vessels, exhibiting tortuous courses and varying calibers
Placental bulge	Same as in 2D
Focal exophytic mass	Same as in 2D
Bridging vessels	Same as in 2D

From Collins et al. [64] with permission.

**Table 4:** MRI sign definitions for PAS disorders.

MRI findings	IS-AIP definition	Sequence type
Heterogeneous placenta	Heterogeneous signal within the placenta	T2W and T1W
Placental bulge	Deviation of the uterine serosa from the expected plane caused by abnormal bulge of placental tissue into neighboring organs (typically the bladder). Uterine serosa appears intact, but outline shape is distorted	T2W and T1W
Dark intraplacental bands	One or more areas of hypointensity with a linear appearance, in contact with the maternal surface of the placenta	T2W
Placental ischemic infarction	Areas of increased signal intensity (T2W) and decreased signal intensity (T1W)	T2W and T1W
Loss of the retroplacental dark zone	Loss of the thin dark zone lying beneath the placental bed	T2W
Myometrial thinning	Thinning of the myometrium overlying the placenta to less than 1 mm or invisible	T2W
Bladder wall interruption	Irregularity or disruption of the normal hypointense urinary bladder wall	T2W
Focal exophytic mass	Placental tissue seen protruding through the uterine wall and extending beyond it. Most often seen inside a filled urinary bladder	T2W and T1W
Placental bed abnormal vascularization	Large vessels within the placental bed with disruption of the uteroplacental interface	T2W

From Morel et al. [70] with permission.

## References

[1] Pattinson R. C., Hall M. (2003). Near misses: a useful adjunct to maternal death enquiries. *British Medical Bulletin*.

[2] Wright J. D., Devine P., Shah M. (2010). Morbidity and mortality of peripartum hysterectomy. *Obstetrics and Gynecology*.

[3] Mesleh R., Ayoub H., Algwiser A., Kurdi A. (1998). Emergency peripartum hysterectomy. *Journal of Obstetrics and Gynaecology*.

[4] Plauché W., Gruich F., Bourgeois M. (1981). Hysterectomy at the time of cesarean section: analysis of 108 cases. *Obstetrics & Gynecology*.

[5] THONET R. G. N. (1986). Obstetric hysterectomy-an 11-year experience. *British Journal of Obstetrics and Gynaecology*.

[6] Huls C. K. (2016). Cesarean hysterectomy and uterine-preserving alternatives. *Obstetrics and Gynecology Clinics of North America*.

[7] Huque S., Roberts I., Fawole B., Chaudhri R., Arulkumaran S., Shakur-Still H. (2018). Risk factors for peripartum hysterectomy among women with postpartum haemorrhage: analysis of data from the WOMAN trial. *BMC Pregnancy Childbirth*.

[8] Schaap T., Bloemenkamp K., Deneux-Tharaux C. (2019). Defining definitions: a Delphi study to develop a core outcome set for conditions of severe maternal morbidity. *BJOG: An International Journal of Obstetrics & Gynaecology*.

[9] Rossi A. C., Lee R. H., Chmait R. H. (2010). Emergency postpartum hysterectomy for uncontrolled postpartum bleeding. *Obstetrics and Gynecology*.

[10] Kallianidis A. F., Maraschini A., Danis J. (2020). Epidemiological analysis of peripartum hysterectomy across nine European countries. *Acta Obstetricia et Gynecologica Scandinavica*.

[11] Bateman B. T., Mhyre J. M., Callaghan W. M., Kuklina E. V. (2012). Peripartum hysterectomy in the United States: nationwide 14 year experience. *American Journal of Obstetrics and Gynecology*.

[12] van den Akker T., Brobbel C., Dekkers O. M., Bloemenkamp K. W. M. (2016). Prevalence, indications, risk indicators, and outcomes of emergency peripartum hysterectomy worldwide. *Obstetrics and Gynecology*.

[13] Sahin S., Guzin K., Eroğlu M., Kayabasoglu F., Yaşartekin M. S. (2014). Emergency peripartum hysterectomy: our 12-year experience. *Archives of Gynecology and Obstetrics*.

[14] Knight M., Kurinczuk J. J., Spark P., Brocklehurst P., United Kingdom Obstetric Surveillance System Steering Committee (2008). Cesarean delivery and peripartum hysterectomy. *Obstetrics and Gynecology*.

[15] Campbell S. M., Corcoran P., Manning E., Greene R. A. (2016). Peripartum hysterectomy incidence, risk factors and clinical characteristics in Ireland. *European Journal of Obstetrics, Gynecology, and Reproductive Biology*.

[16] Kwee A., Bots M. L., Visser G. H. A., Bruinse H. W. (2006). Emergency peripartum hysterectomy: a prospective study in the Netherlands. *European Journal of Obstetrics, Gynecology, and Reproductive Biology*.

[17] Bodelon C., Bernabe-Ortiz A., Schiff M. A., Reed S. D. (2009). Factors associated with peripartum hysterectomy. *Obstetrics and Gynecology*.

[18] Awan N., Bennett M. J., Walters W. A. W. (2011). Emergency peripartum hysterectomy: a 10-year review at the Royal Hospital for women, Sydney. *The Australian & New Zealand Journal of Obstetrics & Gynaecology*.

[19] Whiteman M. K., Kuklina E., Hillis S. D. (2006). Incidence and determinants of peripartum hysterectomy. *Obstetrics and Gynecology*.

[20] Silver R. M., Landon M. B., Rouse D. J. (2006). Maternal morbidity associated with multiple repeat cesarean deliveries. *Obstetrics and Gynecology*.

[21] Wright J. D., Herzog T. J., Shah M. (2010). Regionalization of care for obstetric hemorrhage and its effect on maternal mortality. *Obstetrics and Gynecology*.

[22] Clark E. A. S., Silver R. M. (2011). Long-term maternal morbidity associated with repeat cesarean delivery. *American Journal of Obstetrics and Gynecology*.

[23] World Health Organization (2012). *WHO recommendations for the prevention and treatment of postpartum haemorrhage*.

[24] Maneschi F., Perrone S., Di Lucia A., Ianiri P. (2020). Shock parameters and shock index during severe post-partum haemorrhage and implications for management: a clinical study. *Journal of Obstetrics and Gynaecology*.

[25] El Ayadi A. M., Nathan H. L., Seed P. T. (2016). Vital sign prediction of adverse maternal outcomes in women with hypovolemic shock: the role of shock index. *PLoS One*.

[26] Bonnar J. (2000). Massive obstetric haemorrhage. *Best Practice & Research Clinical Obstetrics & Gynaecology*.

[27] Lee J. Y., Ahn E. H., Kang S. (2018). Scoring model to predict massive post-partum bleeding in pregnancies with placenta previa: a retrospective cohort study. *The Journal of Obstetrics and Gynaecology Research*.

[28] Say L., Chou D., Gemmill A. (2014). Global causes of maternal death: a WHO systematic analysis. *The Lancet Global Health*.

[29] Flood K. M., Said S., Geary M., Robson M., Fitzpatrick C., Malone F. D. (2009). Changing trends in peripartum hysterectomy over the last 4 decades. *American Journal of Obstetrics and Gynecology*.

[30] Morlando M., Collins S. (2020). Placenta accreta spectrum disorders: challenges, risks, and management strategies. *International Journal of Women's Health*.

[31] Jauniaux E., Ayres-de-Campos D., Placenta Accreta Diagnosis F. I. G. O., Panel M. E. C. (2018). FIGO consensus guidelines on placenta accreta spectrum disorders: introduction,. *International Journal of Gynaecology and Obstetrics*.

[32] Jauniaux E., Alfirevic Z., Bhide A. G. (2019). Placenta praevia and placenta accreta: diagnosis and management. *BJOG: An International Journal of Obstetrics & Gynaecology*.

[33] (2018). Obstetric care consensus no. 7 summary: placenta accreta spectrum. *Obstetrics & Gynecology*.

[34] Jauniaux E., Collins S., Burton G. J. (2018). Placenta accreta spectrum: pathophysiology and evidence-based anatomy for prenatal ultrasound imaging. *American Journal of Obstetrics and Gynecology*.

[35] Chantraine F., Braun T., Gonser M., Henrich W., Tutschek B. (2013). Prenatal diagnosis of abnormally invasive placenta reduces maternal peripartum hemorrhage and morbidity. *Acta Obstetricia et Gynecologica Scandinavica*.

[36] Jauniaux E., Chantraine F., Silver R. M., Langhoff-Roos J., Placenta Accreta Diagnosis F. I. G. O., Panel M. E. C. (2018). FIGO consensus guidelines on placenta accreta spectrum disorders: epidemiology. *International Journal of Gynaecology and Obstetrics*.

[37] Jauniaux E., Jurkovic D. (2012). Placenta accreta: pathogenesis of a 20th century iatrogenic uterine disease. *Placenta*.

[38] Jauniaux E., Ayres-de-Campos D., Langhoff-Roos J. (2019). FIGO classification for the clinical diagnosis of placenta accreta spectrum disorders. *International Journal of Gynaecology and Obstetrics*.

[39] Jauniaux E., Grønbeck L., Bunce C., Langhoff-Roos J., Collins S. L. (2019). Epidemiology of placenta previa accreta: a systematic review and meta-analysis. *BMJ Open*.

[40] Modest A. M., Toth T. L., Johnson K. M., Shainker S. A. (2020). Placenta accreta spectrum: in vitro fertilization and non-in vitro fertilization and placenta accreta spectrum in a Massachusetts cohort. *American Journal of Perinatology*.

[41] Salmanian B., Fox K. A., Arian S. E. (2020). In vitro fertilization as an independent risk factor for placenta accreta spectrum. *American Journal of Obstetrics and Gynecology*.

[42] Khong T. Y., Robertson W. B. (1987). Placenta creta and placenta praevia creta. *Placenta*.

[43] Gurol-Urganci I., Cromwell D. A., Edozien L. C. (2011). Risk of placenta previa in second birth after first birth cesarean section: a population-based study and meta-analysis. *BMC Pregnancy and Childbirth*.

[44] Ananth C. V., Smulian J. C., Vintzileos A. M. (1997). The association of placenta previa with history of cesarean delivery and abortion: a metaanalysis. *American Journal of Obstetrics and Gynecology*.

[45] Jauniaux E., Bhide A. (2017). Prenatal ultrasound diagnosis and outcome of placenta previa accreta after cesarean delivery: a systematic review and meta-analysis. *American Journal of Obstetrics and Gynecology*.

[46] Brookfield K. F., Goodnough L. T., Lyell D. J., Butwick A. J. (2014). Perioperative and transfusion outcomes in women undergoing cesarean hysterectomy for abnormal placentation. *Transfusion*.

[47] Green L., Knight M., Seeney F. M. (2016). The epidemiology and outcomes of women with postpartum haemorrhage requiring massive transfusion with eight or more units of red cells: a national cross-sectional study. *BJOG: An International Journal of Obstetrics & Gynaecology*.

[48] Solheim K. N., Esakoff T. F., Little S. E., Cheng Y. W., Sparks T. N., Caughey A. B. (2011). The effect of cesarean delivery rates on the future incidence of placenta previa, placenta accreta, and maternal mortality. *The Journal of Maternal-Fetal & Neonatal Medicine*.

[49] Baldwin H. J., Patterson J. A., Nippita T. A. (2018). Antecedents of abnormally invasive placenta in primiparous women. *Obstetrics and Gynecology*.

[50] Gyamfi-Bannerman C., Gilbert S., Landon M. B. (2012). Risk of uterine rupture and placenta accreta with prior uterine surgery outside of the lower segment. *Obstetrics and Gynecology*.

[51] Jauniaux E., Hussein A. M., Fox K. A., Collins S. L. (2019). New evidence-based diagnostic and management strategies for placenta accreta spectrum disorders. *Best Practice & Research. Clinical Obstetrics & Gynaecology*.

[52] Shamshirsaz A. A., Fox K. A., Salmanian B. (2015). Maternal morbidity in patients with morbidly adherent placenta treated with and without a standardized multidisciplinary approach. *American Journal of Obstetrics and Gynecology*.

[53] Silver R. M., Fox K. A., Barton J. R. (2015). Center of excellence for placenta accreta. *American Journal of Obstetrics and Gynecology*.

[54] Shamshirsaz A. A., Fox K. A., Erfani H. (2017). Multidisciplinary team learning in the management of the morbidly adherent placenta: outcome improvements over time. *American Journal of Obstetrics and Gynecology*.

[55] Bartels H. C., Rogers A. C., O’Brien D., McVey R., Walsh J., Brennan D. J. (2018). Association of implementing a multidisciplinary team approach in the management of morbidly adherent placenta with maternal morbidity and mortality. *Obstetrics and Gynecology*.

[56] Silver R. M., Branch D. W. (2018). Placenta accreta spectrum. *The New England Journal of Medicine*.

[57] Fitzpatrick K. E., Sellers S., Spark P., Kurinczuk J. J., Brocklehurst P., Knight M. (2014). The management and outcomes of placenta accreta, increta, and percreta in the UK: a population-based descriptive study. *BJOG: An International Journal of Obstetrics & Gynaecology*.

[58] Buca D., Liberati M., Calì G. (2018). Influence of prenatal diagnosis of abnormally invasive placenta on maternal outcome: systematic review and meta-analysis. *Ultrasound in Obstetrics & Gynecology*.

[59] Bailit J. L., Grobman W. A., Rice M. M. (2015). Morbidly adherent placenta treatments and outcomes. *Obstetrics and Gynecology*.

[60] Bowman Z. S., Eller A. G., Bardsley T. R., Greene T., Varner M. W., Silver R. M. (2014). Risk factors for placenta accreta: a large prospective cohort. *American Journal of Perinatology*.

[61] Thurn L., Lindqvist P. G., Jakobsson M. (2016). Abnormally invasive placenta-prevalence, risk factors and antenatal suspicion: results from a large population-based pregnancy cohort study in the Nordic countries. *BJOG: An International Journal of Obstetrics & Gynaecology*.

[62] Jauniaux E., Bhide A., Kennedy A. (2018). FIGO consensus guidelines on placenta accreta spectrum disorders: prenatal diagnosis and screening. *International Journal of Gynaecology and Obstetrics*.

[63] D’Antonio F., Iacovella C., Bhide A. (2013). Prenatal identification of invasive placentation using ultrasound: systematic review and meta-analysis. *Ultrasound in Obstetrics & Gynecology*.

[64] Collins S. L., Ashcroft A., Braun T. (2016). Proposal for standardized ultrasound descriptors of abnormally invasive placenta (AIP). *Ultrasound in Obstetrics & Gynecology*.

[65] Jauniaux E., Collins S. L., Jurkovic D., Burton G. J. (2016). Accreta placentation: a systematic review of prenatal ultrasound imaging and grading of villous invasiveness. *American Journal of Obstetrics and Gynecology*.

[66] Familiari A., Liberati M., Lim P. (2018). Diagnostic accuracy of magnetic resonance imaging in detecting the severity of abnormal invasive placenta: a systematic review and meta-analysis. *Acta Obstetricia et Gynecologica Scandinavica*.

[67] Meng X., Xie L., Song W. (2013). Comparing the diagnostic value of ultrasound and magnetic resonance imaging for placenta accreta: a systematic review and meta-analysis. *Ultrasound in Medicine & Biology*.

[68] Panaiotova J., Tokunaka M., Krajewska K., Zosmer N., Nicolaides K. H. (2019). Screening for morbidly adherent placenta in early pregnancy. *Ultrasound in Obstetrics & Gynecology*.

[69] Einerson B. D., Rodriguez C. E., Kennedy A. M., Woodward P. J., Donnelly M. A., Silver R. M. (2018). Magnetic resonance imaging is often misleading when used as an adjunct to ultrasound in the management of placenta accreta spectrum disorders. *American Journal of Obstetrics and Gynecology*.

[70] Morel O., Collins S. L., Uzan-Augui J. (2019). A proposal for standardized magnetic resonance imaging (MRI) descriptors of abnormally invasive placenta (AIP)–from the International Society for AIP. *Diagnostic and Interventional Imaging*.

[71] Gielchinsky Y., Rojansky N., Fasouliotis S. J., Ezra Y. (2002). Placenta accreta—summary of 10 years: a survey of 310 cases. *Placenta*.

[72] Sheiner E., Levy A., Katz M., Mazor M. (2003). Identifying risk factors for peripartum cesarean hysterectomy. A population-based study. *The Journal of Reproductive Medicine*.

[73] Bencaiova G., Burkhardt T., Beinder E. (2007). Abnormal placental invasion experience at 1 center. *The Journal of Reproductive Medicine*.

[74] Klar M., Laub M., Schulte-Moenting J., Proempeler H., Kunze M. (2013). Clinical risk factors for complete and partial placental retention - a case-control study. *Journal of Perinatal Medicine*.

[75] Woodring T. C., Klauser C. K., Bofill J. A., Martin R. W., Morrison J. C. (2011). Prediction of placenta accreta by ultrasonography and color Doppler imaging. *The Journal of Maternal-Fetal & Neonatal Medicine*.

[76] Collins S. L., Alemdar B., van Beekhuizen H. (2019). Evidence-based guidelines for the management of abnormally invasive placenta: recommendations from the International Society for Abnormally Invasive Placenta. *American Journal of Obstetrics and Gynecology*.

[77] Irving C., Hertig A. (1937). A study of placenta accreta. *Surgery, Gynecology and Obstetrics archives*.

[78] Jauniaux E., Bunce C., Grønbeck L., Langhoff-Roos J. (2019). Prevalence and main outcomes of placenta accreta spectrum: a systematic review and meta-analysis. *American Journal of Obstetrics and Gynecology*.

[79] Bartels H. C., Postle J. D., Downey P., Brennan D. J. (2018). Placenta accreta spectrum: a review of pathology, molecular biology, and biomarkers. *Disease Markers*.

[80] Wright J. D., Silver R. M., Bonanno C. (2013). Practice patterns and knowledge of obstetricians and gynecologists regarding placenta accreta. *The Journal of Maternal-Fetal & Neonatal Medicine*.

[81] Esakoff T. F., Handler S. J., Granados J. M., Caughey A. B. (2012). PAMUS: placenta accreta management across the United States. *The Journal of Maternal-Fetal & Neonatal Medicine*.

[82] O’Brien J. M., Barton J. R., Donaldson E. S. (1996). The management of placenta percreta: conservative and operative strategies. *American Journal of Obstetrics and Gynecology*.

[83] Melcer Y., Jauniaux E., Maymon S. (2018). Impact of targeted scanning protocols on perinatal outcomes in pregnancies at risk of placenta accreta spectrum or vasa previa. *American Journal of Obstetrics and Gynecology*.

[84] Amsalem H., Kingdom J. C. P., Farine D. (2011). Planned caesarean hysterectomy versus “conserving” caesarean section in patients with placenta accreta. *Journal of Obstetrics and Gynaecology Canada*.

[85] Chantraine F., Langhoff-Roos J. (2013). Abnormally invasive placenta - AIP. Awareness and pro-active management is necessary. *Acta Obstetricia et Gynecologica Scandinavica*.

[86] Pather S., Strockyj S., Richards A., Campbell N., de Vries B., Ogle R. (2014). Maternal outcome after conservative management of placenta percreta at caesarean section: a report of three cases and a review of the literature. *The Australian & New Zealand Journal of Obstetrics & Gynaecology*.

[87] Matsuzaki S., Yoshino K., Endo M., Kakigano A., Takiuchi T., Kimura T. (2018). Conservative management of placenta percreta. *International Journal of Gynaecology and Obstetrics*.

[88] Matsubara S. (2012). Planned caesarean hysterectomy versus “conserving” caesarean section in patients with placenta accreta. *Journal of Obstetrics and Gynaecology Canada*.

[89] Bazer F., First N. (1983). Pregnancy and parturition. *Journal of Animal Science*.

[90] Palmer S., Zamudio S., Coffin C., Parker S., Stamm E., Moore L. (1992). Quantitative estimation of human uterine artery blood flow and pelvic blood flow redistribution in pregnancy. *Obstetrics and gynaecology*.

[91] Allen L., Jauniaux E., Hobson S. (2018). FIGO consensus guidelines on placenta accreta spectrum disorders: nonconservative surgical management,. *International Journal of Gynaecology and Obstetrics*.

[92] Smaill F. M., Grivell R. M. (2014). Antibiotic prophylaxis versus no prophylaxis for preventing infection after cesarean section. *Cochrane Database of Systematic Reviews*.

[93] Shakur H., Roberts I., Fawole B. (2017). Effect of early tranexamic acid administration on mortality, hysterectomy, and other morbidities in women with post-partum haemorrhage (WOMAN): an international, randomised, double-blind, placebo-controlled trial. *The Lancet*.

[94] Rossetti D., Vitale S. G., Bogani G., Rapisarda A. M. C., Gulino F. A., Frigerio L. (2015). Usefulness of vessel-sealing devices for peripartum hysterectomy: a retrospective cohort study. *Updates in Surgery*.

[95] Eller A. G., Porter T. F., Soisson P., Silver R. M. (2009). Optimal management strategies for placenta accreta. *BJOG: An International Journal of Obstetrics & Gynaecology*.

[96] Gyamfi-Bannerman C., Thom E. A., Blackwell S. C. (2016). Antenatal betamethasone for women at risk for late preterm delivery. *The New England Journal of Medicine*.

[97] Hoffman M. S. (2010). Morbidity associated with nonemergent hysterectomy for placenta accreta. *American Journal of Obstetrics and Gynecology*.

[98] Sentilhes L., Goffinet F., Kayem G. (2013). Management of placenta accreta. *Acta Obstetricia et Gynecologica Scandinavica*.

[99] al-Khan A., Gupta V., Illsley N. P. (2014). Maternal and fetal outcomes in placenta accreta after institution of team-managed care. *Reproductive Sciences*.

[100] Upson K., Silver R. M., Greene R., Lutomski J., Holt V. L. (2014). Placenta accreta and maternal morbidity in the Republic of Ireland, 2005-2010. *The Journal of Maternal-Fetal & Neonatal Medicine*.

[101] Tam Tam K. B., Dozier J., Martin J. N. (2012). Approaches to reduce urinary tract injury during management of placenta accreta, increta, and percreta: a systematic review. *The Journal of Maternal-Fetal & Neonatal Medicine*.

[102] Silver R. M. (2015). Abnormal Placentation. *Obstetrics & Gynecology*.

[103] Woldu S. L., Ordonez M. A., Devine P. C., Wright J. D. (2014). Urologic considerations of placenta accreta: a contemporary tertiary care institutional experience. *Urologia Internationalis*.

[104] Papillon-Smith J., Singh S. S., Ziegler C. (2016). Internal iliac artery rupture caused by endovascular balloons in a woman with placenta percreta. *Journal of Obstetrics and Gynaecology Canada*.

[105] Shrivastava V., Nageotte M., Major C., Haydon M., Wing D. (2007). Case-control comparison of cesarean hysterectomy with and without prophylactic placement of intravascular balloon catheters for placenta accreta. *American Journal of Obstetrics and Gynecology*.

[106] Chen M., Liu X., You Y. (2020). Internal iliac artery balloon occlusion for placenta previa and suspected placenta accreta. *Obstetrics and Gynecology*.

[107] Salim R., Chulski A., Romano S., Garmi G., Rudin M., Shalev E. (2015). Precesarean prophylactic balloon catheters for suspected placenta accreta. *Obstetrics and Gynecology*.

[108] Hussein A. M., Dakhly D. M. R., Raslan A. N. (2019). The role of prophylactic internal iliac artery ligation in abnormally invasive placenta undergoing caesarean hysterectomy: a randomized control trial. *The Journal of Maternal-Fetal & Neonatal Medicine*.

[109] Matsubara S., Kuwata T., Usui R. (2013). Important surgical measures and techniques at cesarean hysterectomy for placenta previa accreta. *Acta Obstetricia et Gynecologica Scandinavica*.

[110] Kingdom J. C., Hobson S. R., Murji A. (2020). Minimizing surgical blood loss at cesarean hysterectomy for placenta previa with evidence of placenta increta or placenta percreta: the state of play in 2020. *American Journal of Obstetrics and Gynecology*.

[111] Shih J.-C., Liu K.-L., Shyu M.-K. (2005). Temporary balloon occlusion of the common iliac artery: new approach to bleeding control during cesarean hysterectomy for placenta percreta. *American Journal of Obstetrics and Gynecology*.

[112] Matsubara S., Kuwata T., Usui R. (2011). Forceps holding the cervix for postpartum haemorrhage. *Journal of Obstetrics and Gynaecology*.

[113] Matsubara S., Ohkuchi A., Yashi M. (2009). Opening the bladder for cesarean hysterectomy for placenta previa percreta with bladder invasion. *The Journal of Obstetrics and Gynaecology Research*.

[114] Burchell R. C. (1967). Arterial blood flow into the human intervillous space. *American Journal of Obstetrics and Gynecology*.

[115] Murji A., Kingdom J. (2019). Placenta percreta involving maternal bladder. *New England Journal of Medicine*.

[116] Borekci B., Ingec M., Kumtepe Y., Gundogdu C., Kadanali S. (2008). Difficulty of the surgical management of a case with placenta percreta invading towards parametrium. *The Journal of Obstetrics and Gynaecology Research*.

[117] Perel P., Roberts I., Ker K. (2013). Colloids versus crystalloids for fluid resuscitation in critically ill patients. *Cochrane Database of Systematic Reviews*.

